# Multiloop edgewise archwire technique and denture frame analysis: a systematic review

**DOI:** 10.1186/s13005-020-00247-x

**Published:** 2020-11-26

**Authors:** M. Tabancis, A. Ratzmann, P. Doberschütz, K. F. Krey

**Affiliations:** Department of Orthodontics and Craniofacial Orthopedics, University Medicine Greifswald, Greifswald, Germany

**Keywords:** Multiloop edgewise archwire, MEAW, Denture frame analysis, Kim, Sato, Dental compensation

## Abstract

**Background:**

The Multiloop Edgewise Archwire (MEAW) appliance is an orthodontic treatment method suitable for the therapy of severe types of malocclusions such as open bites or anterior crossbites. The cephalometric Denture Frame Analysis (DFA) provides a supportive diagnostic tool for patient-specific treatment planning concerning the rearrangement of occlusion within the “denture frame”. The objective of this study is to give a comprehensive overview of the national and international scientific literature about MEAW and DFA regarding the general therapeutic effects, advantages and limitations.

**Methods and materials:**

A computerized literature search was performed using four principal medical databases (PubMed/Medline, Google Scholar, Web of Science and Cochrane Central Register of Controlled Trials) and supplemented by manual searching of the references listed in the retrieved articles. The results were screened and assessed following the PRISMA guidelines.

**Results:**

Six hundred seventy-seven full articles were assessed for eligibility. A number of 134 articles went through qualitative analysis and 3 studies were finally involved in comparative synopsis. The findings reveal advantageous characteristics of the MEAW technique such as a high degree of three-dimensional individual tooth control and a comparatively low load deflection rate, causing mostly dentoalveolar changes without significantly influencing the skeletal structures.

**Conclusion:**

Based on current literature, the MEAW technique appears to have several therapeutic benefits and serves as a sufficient alternative treatment method for dentoalveolar compensation, when measures of orthognathic surgery are rejected. Concerning the deficient data basis of available literature and the low level of scientific evidence, further studies are required in order to expand on the knowledge in this subject area. Several aspects like the effectiveness or the long-term stability have to be evaluated more extensively. Moreover, the transferability of the DFA to ethnic groups other than the Asian ethnicity should be examined further.

## Background

In 1967, Young H. Kim introduced a treatment philosophy based on anthropological considerations regarding the verticalization of the facial skull during the development of the upright walk of *Homo sapiens* [1]. The changes in the inclination of the occlusal plane that have occurred in this evolutionary context can also be observed in the individual development of man [1, 2]. The multiloop edgewise archwire (MEAW) technique was originally designed for the treatment of open bite patients and has rapidly obtained popularity in the treatment of several other malocclusions [3]. Its underlying concept is based on the hypothesis that selective changes in the inclination of the occlusal plane can compensate various types of malocclusions by utilizing the adaptability of the temporomandibular joint [3]. Sadao Sato from Kanawaga Dental College of Japan acquired and publicized the MEAW concept further [4].

The preparation and activation of this appliance is complex, but offers a maximum of flexibility and control to the practitioner. MEAW arches are made of 0.016″× 0.022″ steel wire with an ideal arch shape, in which five L- loops are incorporated in each quadrant starting distally of the lateral teeth. Kim originally recommended a bracket slot size of 0.018″ for individual torque control [5–8]. The reconstruction of the entire occlusal plane is implemented by influencing the angulation of the posterior teeth by means of tip-back activations of three to five degrees on each tooth [5, 6, 9]. Compensatory forces are exerted through the additional use of intermaxillary elastics [5, 10, 11].

The theory supported by Kim and Sato states that the vertical position and the inclination of the posterior occlusal plane have a direct impact on the anteroposterior position of the mandible and can therefore be associated to particular types of malocclusions [1, 12–17].

A steep posterior occlusal plane is a common finding in skeletal class II cases, while a flat posterior occlusal plane is predominating in class III cases [18, 19]. In order to treat these cases the vertical inclination of the posterior region has to be corrected by flattening or steepening the occlusal plane respectively [12]. These inherent vertical deviations are assumed to be based on a evolutionarily imbalance of the size of the entire alveolar base and the size of all teeth leading to the so-called “molar-crowding” that is enhanced by the collision of erupting third molars and the “squeeze-out” of the adjacent molars [19]. Because of this, the main measures of MEAW therapy are the elimination of the posterior crowding and the uprighting of mesially inclined posterior teeth as well as the reconstruction of the occlusal plane [1, 11, 20], implemented by corresponding activations of the MEAW appliance.

The Denture Frame Analysis (DFA), which was established in 1987, can be used to evaluate the skeletal situation in order to allow the rearrangement of occlusion within the limits of a given “denture frame” [7, 20, 21]. DFA is based on correlations of cephalometric parameters with the values of overbite and overjet. In contrast to the currently widespread straight-wire systems, which are based on average values, MEAW and DFA therefore offer measures of patient-individualized treatment planning and implementation.

The aim of this study was to systematically evaluate the available scientific literature and all publications about the Multiloop Edgewise Archwire technique and the Denture Frame Analysis concerning the therapeutic effects, general cephalometric changes, advantages, disadvantages and limits of MEAW.

## Methods

### Search strategy and study selection

A thorough electronic database search using PubMed, Google Scholar, Web of Science and Cochrane Central Register of Controlled Trials was conducted for publications on the Multiloop Edgewise Archwire technique and the Denture Frame Analysis. As there was no correspondance to any MeSH terms, the following single terms were used in free-text search: “MEAW”, “multiloop”, “multi-loop”, “Sato technique”, “edgewise”, “denture frame”, “Kim analysis cephalometrics”, “MOAW”, “modified offset archwire”, “SMOM”, “sectional modified offset archwire”. For completeness, an additional manual search was carried out. No restrictions regarding date of publication were set.

Open access publications and papers published in journals via license of Greifswald University in the English, German, French, Italian, Portuguese, Spanish and Greek language were included in the review. To ensure that the treatment effects described are based only on the effects of MEAW, studies implementing additional means of skeletal anchorage were excluded from consideration. The time period chosen for the online search strategy was from mid-march to mid-april 2020.

Data extraction was performed by two researchers (MT, KFK). The results have been screened and assessed following the predetermined eligibility criteria and arranged according to the Preferred Reporting Items for Systematic Reviews and Meta-Analyses (PRISMA) guidelines [22].

### Data items

Information retrieved from included the type of study, the number of subjects, the gender distribution as well as the following aspects of MEAW therapy:

1. Therapeutic effects
2. Denture Frame Analysis:
  - General cephalometric changes
  - Cephalometric changes according to study results
3. Advantages
4. Disadvantages
5. Limits

## Results

### Data selection

**Fig. 1:**
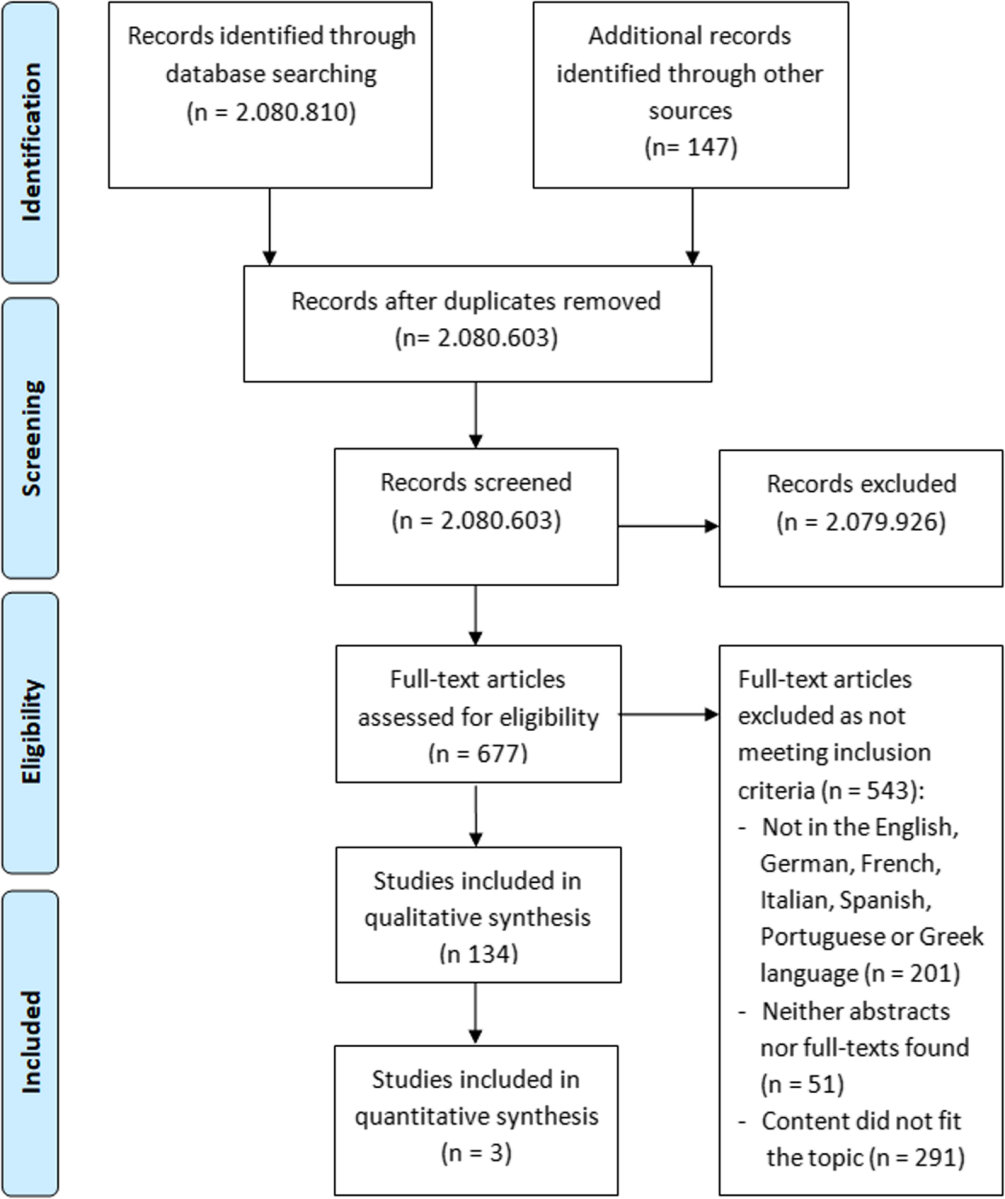
Flow diagram showing the study selection process according to PRISMA guidelines

Since only single terms were used in search of online databases a high total number of results (2.080.810) was retrieved. In the course of literature search, all found abstracts were examined with regard to their correspondence to the subject. Subsequently, the literature cited in the references was screened for additional significant articles meeting the inclusion criteria. A total of 677 full-texts were assessed for eligibility out of which 543 articles were excluded due to non-fulfilment of the predetermined criteria. Finally, 134 full articles were included in a qualitative synthesis. After a thorough evaluation of all retrieved publications, 3 studies [7, 20, 23] with common cephalometric values were involved in our study. The detailed arrangement according to PRISMA-guidelines is presented in Fig. 1.

The evidence level of each study was assessed according to the Oxford Centre for Evidence-Based Medicine criteria [24]. According to this, we found 1 randomized controlled trial, 2 non-randomized clinical trials, 2 case-control studies, 12 comparative studies, 17 descriptive studies, 67 case reports, 7 literature review articles, handout material from 8 scientific lectures, 2 articles reflecting expert opinions and 16 articles with background information. All studies had a low evidence level. Based on these publications we collected general information about the principles of the MEAW technique as well as the effects, the limits, the advantages and disadvantages of this technique as presented below.

### General therapeutic effects

The focus of the MEAW technique lies in the control of the occlusal vertical dimension. The main therapeutic changes have been observed to occur in the dentoalveolar region, while only minor skeletal effects have been discovered [11, 15, 25, 26]. In treatments using MEAW a mesiodistal tipping movement causes a lower amount of individual tooth displacement and more uniform distribution of stress compared to treatments with the Ideal Achwire (IA) without loops [6, 14]. In contrast to the posterior teeth, the labiolingual tipping movements in the anterior region is higher with MEAW, but yet lower than with the IA [6].

The anterior vertical elastics act cooperatively with the MEAW appliance and convert the forces resulting from the tipback bends into the desired movements [11]. Thereby, the intrusive forces on the anterior teeth are counteracted through the extrusive dynamics of the vertical elastics, inducing lingual crown tipping and bodily displacement of the anterior teeth [6, 27]. The posterior teeth are subjected to distal uprighting [7, 27, 28]. In class II cases, extrusion of the maxillary premolars and molars causes flattening of the occlusal plane and thereby allows the forward movement of the mandible [29]. In contrast, in class III cases, the extrusion of the upper molars accompanied by the extrusion of the lower premolars and molars results in a steeper occlusal plane, so that the mandible can move backward [3, 7, 30]. Moreover, MEAW was found to be a very effective tool in the en-masse retraction of the mandibular dentition in class III patients [6].

According to Endo et al. [7] the correction of open bite cases is attained through the extrusion and uprighting of the posterior teeth resulting in an upward and forward rotation of the mandible. This is supported by downward vertical forces exerted by intermaxillary elastics causing the mandibular ramus to shift downward. The incisors are extruded, uprighted and can be retruded when indicated. The extrusion of all canines ensures a cuspid-protected occlusion [7].

*Yoshimura* et al [17] compared the treatment effects of the MEAW technique with those of the IA including the use of vertical elastics. According to their results, the molars, premolars and canines can be successfully uprighted and rotated distally by means of MEAW. With the IA, this effect was found only in the second molars. A very light mesial inclining and distal extrusive force was observed in the first molars, while IA has exerted only extrusive forces on premolars and canines. Both techniques induced extrusion of the lateral incisors, while mesial inclination was also detected when using the IA [17]. These findings underline the unique ability of the MEAW appliance in uprighting mesially inclined teeth due to the special construction of multiple loops.

### Denture frame analysis

#### Cephalometric changes

In addition to the MEAW technique, *Sato* introduced the DFA [30, 31], a special cephalometric analysis. The main idea of his philosophy is to rearrange the malpositioned teeth within the individual skeletal frame of each patient [32]. This triangular frame is built by the A-B plane, the palatal plane and the mandibular plane [30, 33]. One of the primary goals is the determination of vertical and horizontal growth patterns in relation to the occlusal plane [30].

There are three characteristic values: Overbite Depth Indicator (ODI), Anteroposterior Dysplasia Index (APDI) and Combination Factor (CF). The ODI is used to characterize the vertical growth pattern by assessing the inclination of the maxillary and the mandibular base and by expressing their relationship in the horizontal dimension [31, 32, 34, 35]. The APDI determines the sagittal jaw base relationship [15, 30, 34, 36–38] and is defined as arithmetic sum of three angles: the Frankfurt horizontal plane to the face plane, the AB plane to the face plane and the Frankfurt horizontal plane to the palatal plane [37, 38]. The CF is obtained by the sum of ODI and APDI combining vertical and horizontal values and it represents a useful tool in the extraction or non-extraction decision [29, 39].

In the scientific literature, only a few publications [7, 11, 14, 15, 20, 23, 40, 41] were found that reproduce the therapeutic effects of the MEAW appliance by means of initial and final cephalometric records and the DFA. The differences have been mainly detected in the dentoalveolar region, while the skeletal measurements have remained almost unchanged [11, 20]. The study results by *Chang and Moon* [20] only recorded an increase of ODI, which was due to changes of the AB-plane and the mandibular position. Significant changes in the position of the upper and lower second molars in relation to the pterygomaxillary fissure demonstrate the successful distal movement of the whole dentition. Regarding this, the literature shows that the implement of distal uprighting as well as intrusion or extrusion of the posterior teeth is feasible with the MEAW appliance [20]. In addition, the distal tipping movement of upper and lower premolars with MEAW was found to be more significant than in the molars [20]. This tipping movement in turn helps in the overjet correction by gaining space for retraction of anterior teeth [14]. Moreover, the cephalometric changes of the OP-MP angle indicate the effective modifications made to the inclination of the occlusal plane by controlling the vertical dimension with MEAW, which is one of the main aims of this treatment modality [15, 20]. These results are contrary to the findings of *Liu* et al.*,* who did not identify significant changes of the occlusal plane level [41]. These discrepancies in study results emphasize the need of further investigations with representative numbers of subjects in order to be able to obtain clear statements.

*Endo* et al. [7] confirm the findings by *Chang and Moon*: The dentoalveolar values have shown a successful increase in overbite and also in the interincisal angle suggesting the extrusion and retroclination of upper and lower anteriors. This in turn has affected soft tissues leading to the lower lip retraction with increased anterior tooth display. In the maxillary and mandibular dentition the canines, the premolars and the molars have been successfully uprighted and retruded [7]. Aditionally, the upper second molars have been noticeably extruded in order to flatten the posterior occlusal plane. The successful uprighting of mesially inclined posterior teeth has also been reported in further studies [11, 23].

Regarding skeletal changes, the point B and pogonion have moved towards the y-axis as a result of open bite correction. Moreover, the ramus inclination was found to have increased by 1.4° [7]. Except of the altered RL-SN angle, no other significant skeletal changes have been detected during the MEAW treatment [7]. The downward and forward rotation of the mandible was no result of growth change, but it has been induced by the control of inclination of the occlusal plane. These assessments coincide with the results of two further studies by *Koncz* et al. and by *Kim* et al., where no significant changes have been discovered in any of the skeletal values [11, 23]. *Krey and Dannhauer* have also found no changes in the basic skeletal pattern of class II [40]. This implies that the rearrangement of the dentition with the MEAW takes place within the given framework.

Contradictory statements are found regarding changes in the vertical dimension. The study by *Kim* et al. showed an increase in anterior and posterior lower facial height as well as the anterior total facial height [23]. *Endo* et al. found no alterations in the separate facial heights but only in the total posterior facial height [7], while *Krey and Dannhauer* did not detect any vertical differences [40].

#### Comparative evaluation of cephalometric changes

**Fig. 2:**
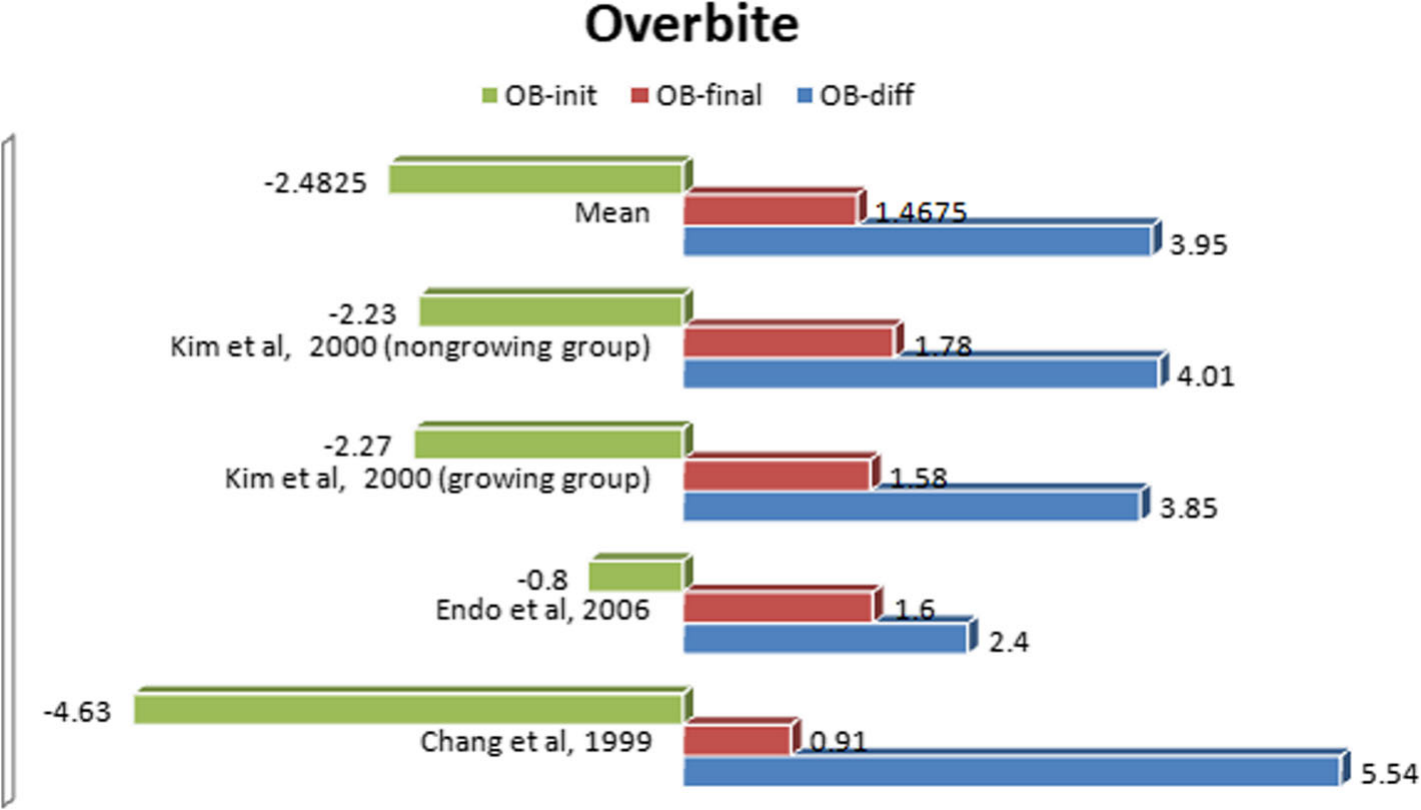
A comparative synopsis of the overbite before and after MEAW treatment

**Fig. 3:**
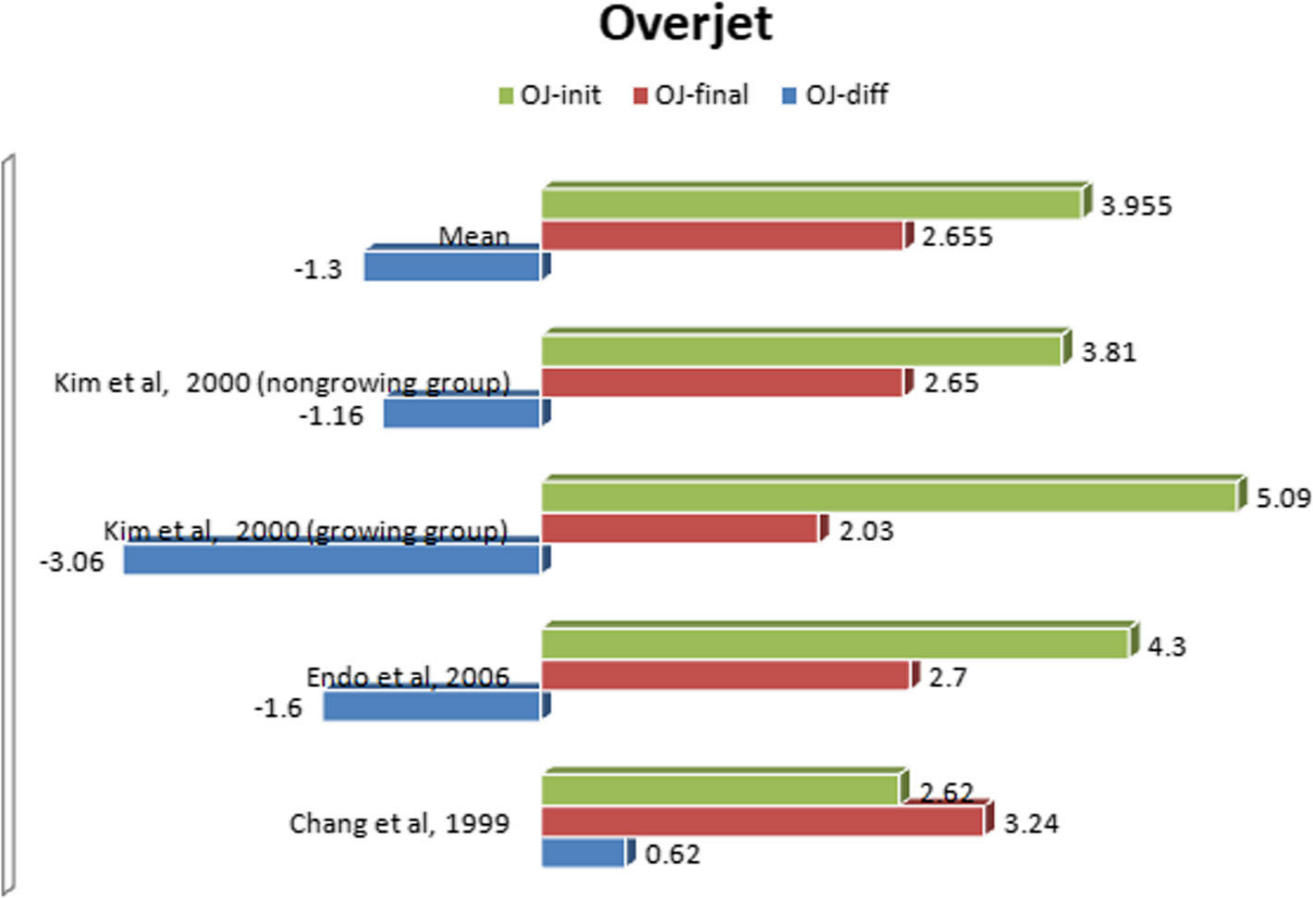
A comparative synopsis of the therapeutic changes of the overjet as a result of MEAW treatment

After a detailed evaluation of all available publications, we identified three studies [7, 20, 23] assessing the same cephalometric values. *Kim* and *Sato* emphasized the fundamental role of interincisal relationship in ensuring stability of treatment results. Therefore, our analysis focused on the treatment-related changes of overbite and overjet before and after MEAW treatment (Figs. 2 and 3). Moreover, the studies were evaluated for design, subject information and level of evidence in accordance with the guidelines of Oxford Centre for Evidence-Based Medicine [24] and are listed below in tabular form (Tab. 1).

**Table 1:** Overview of the relevant articles

Authors	Study design	Evidence level	N	Mean age in years	Item
Kim et al. (2000) [23]	Follow-up Study	3	29	13.5	Overbite and overjet before and after MEAW-treatment
			26	26.1	
Endo et al. (2006) [7]	Follow-up Study	3	21	16.9	
Chang et al. (1999) [20]	Case-Control Study	4	16	18.1	

The study by *Kim* et al. examined 29 growing Eurasian subjects (21 females, 8 males) and 26 non-growing Eurasian subjects (21 females, 5 males) who presented with anterior open bite [23]. At the start of treatment, the mean age of the growing group was 13 years 5 months (range 10–16 years of age), while the average age in the non-growing group was 26 years 1 month (range 17–37 years of age). In the growing group, pretreatment records showed a mean overbite of − 2.27 mm with a standard deviation (SD) of ±2.10 mm and a mean overjet of 5.09 mm (SD ±2.45 mm). After MEAW treatment, a mean overbite of 1.58 mm (SD ±0.81 mm) and a mean overjet of 2.03 mm (SD ±0.69 mm) were documented. All in all, the overbite improved by 3.31 mm and the overjet by 3.06 mm. In the non-growing group, the pretreatment records showed a mean overbite of − 2.23 mm (SD ±2.10 mm) and a mean overjet of 3.81 mm (SD ±2.58 mm). The posttreatment records exposed a mean overbite of 1.78 mm (SD ±0.84 mm) and a mean overjet of 2.65 mm (SD ±0.60 mm). Overall, the overbite was optimized by 3.55 mm and the overjet by 1.16 mm. The results of both groups reflect the efficiency of the MEAW technique in correcting open bite situation as well as enlarged overjet.

*Endo* et al. also performed orthodontic correction on 21 Japanese subjects with anterior open bite using the MEAW technique [7]. The subjects were all female and had a mean pre-treatment age of 16 years 9 months. The final cephalometric records demonstrated an improvement of overbite and overjet by 0.8 mm and 1.6 mm respectively.

In the investigation conducted by *Chang and Moon*, 16 South Korean patients with open bite and MEAW treatment were examined in direct comparison with 58 controls [20]. The initial mean age of the 12 female and 4 male patients was 18 years 1 month. After MEAW therapy, a significant improvement of the initial negative overbite of 5.54 mm was recorded. The average overjet changed by 0.62 mm.

The results of these three studies indicate that the MEAW appliance has a direct therapeutic impact on the overbite and overjet. The necessary space for these sagittal and vertical corrections was obtained by uprighting the mesially inclined posterior teeth. Fifteen degrees of molar uprighting gains as much space as 4.5 mm of distalization [39]. Overall, the literature states that the MEAW technique mainly affects dental changes, while the skeletal components remain largely unchanged. This reinforces the idea of MEAW being more of a camouflage treatment method.

#### Advantages

According to several investigations, the movement of teeth with MEAW is more uniform with a fairly balanced stress distribution through the entire dentition [6, 14, 27]. The increased wire length and the multiple loops induce a high grade of flexibility and reduce the load deflection rate (LDR) down to 1/10 of the LDR found in the IA, thus providing gentle but continual orthodontic forces for biologically advantageous tooth movement [5, 17, 19, 27, 42–44]. Due to the wire length of the horizontal loops increasing from anterior to posterior, the LDR values are high in the anterior segment and low in the posterior segments [6, 42]. The average length of the wire from the distal end of the lateral incisor bracket to the second molar tube amounts 43 mm for a flat NiTi wire and 120 mm for the MEAW [42]. The loops act as stress breakers and exert the optimal amount of force for physiologic orthodontic tooth movements [29, 42, 44]. In addition, the single wire segments enable the individual tooth movement with three-dimensional control through the vertical and horizontal components of the loops [6, 9, 11, 16, 17]. Compared with other materials, the LDR of the MEAW is stiffer than a TMA wire and twice as stiff as a NiTi wire. Yet due to its special construction, the LDR varies from region to region and thus provides unique mechanical properties [42, 43]. Measured in the interbracket region, the loops of the MEAW have a lower LDR than TMA and NiTi wires, which corresponds to Kim’s estimate [27, 43]. This can be traced back to the fact that the horizontal component of the L-loops affects the vertical elastic deflection and consequently reduces the stiffness.

Concerning Class III cases, the MEAW technique appears to have therapeutic advantages making the en-masse movement of the mandibular dentition easier [44, 45]. Concerning this, only minimal amount of vertical displacement of the posterior teeth has been detected with the MEAW during en-masse retraction, which underlines the fact that side effects like extrusive vertical tooth displacement of the anterior teeth are minimized and thus a higher degree of stability is guaranteed than with the application of the IA [14]. These findings agree with the study results received by *Chang* et al. [6]

Following detailed diagnostic analysis, the distal uprighting of posterior teeth can provide enough space to facilitate a treatment without premolar extractions [7]. Moreover, the simultaneous movement of all teeth significantly reduces the overall treatment duration [5, 15, 17, 20, 46] and it serves as an effective alternative treatment method of severe malocclusions avoiding measures of orthognathic surgery [10, 40, 47].

#### Disadvantages

For effective orthodontic treatment with the MEAW technique, the orthodontist needs to have a good knowledge of this method as well as good bending skills. The implementation of the large number of bends requires precise execution in order to achieve the planned dental movements and to avoid side effects [14, 27, 39]. No patient complaints were referred regarding the use of the MEAW appliance. On the contrary, positive feedback was given concerning less toothache and a more comfortable feeling [17]. However, it can be assumed that the measures of hygiene are more complicated and time-consuming due to the large number of loops.

As with numerous other orthodontic treatment devices, the patient’s compliance is essential. Vertical elastics have to be worn permanently in order to achieve the desired results e.g. the extrusion of the anterior teeth, otherwise unwanted intrusion side effects can be caused instead due to the tipback bends [11].

#### Limits

While comparing the MEAW method with the straight wire technique some limiting factors occur. The main treatment changes were evident in the dentoalveolar region and only a few minor skeletal changes have been detected [7, 11, 15, 20, 23, 26, 48]. This underlines the consideration that the MEAW technique is merely a compensatory treatment method as it has no significant impact on the skeletal structure. The induced changes are comparable with natural dentoalveolar compensation [20].

Although the MEAW appliance itself does not represent a risk factor, when used in combination with elastics for a prolonged period of time, the risk of root resorption is increased. If the application period exceeds 6 months, the incidence of root resorptions is the most severe [49]. Long-term “jiggling” may enhance root resorption especially on the anterior teeth, but this hypothesis needs to be scientifically investigated more thoroughly.

Another significant aspect concerns long-term stability. Regarding this aspect, only a few studies could be found in the international scientific literature. MEAW treatment has been proven to achieve successful and stable results in open bite malocclusion [7, 20, 23, 50]. *Rochester* et al. followed up on patients after the completion of the MEAW treatment in order to assess stability. Most changes presented a tendency to relapse right after the treatment, but remained significantly stable after 2 years [26].

Finally, as already mentioned, there is an elementary need for high patient compliance in order to obtain the accurate implementation of the MEAW technique and to avoid undesired side effects [11].

## Discussion

The MEAW technique possesses several mechanical properties that facilitate the correction of various types of malocclusion. It offers the possibility of individual tooth control by the application of gentle and uniform forces. Challenging cases like open bite situations or skeletal class III malocclusions with low to moderate severity can be effectively treated with this method [7, 23, 26, 51].

In contrast to the continual trend towards technological development, the manufacture of the MEAW appliance is more attractive as extensive computer-aided planning and complex additional elements can be dispensed.

Nevertheless, the findings state that the MEAW therapy has only a minimal impact on the basic skeletal patterns. *Chang* et al have observed that the cephalometric values of the MEAW patient group are very close to those of the control group suggesting that the changes induced by the MEAW appliance are comparable to the natural dentoalveolar compensatory mechanism [20].

Due to treatment-related changes predominantly occurring in the dentoalveolar region, MEAW serves as a camouflage treatment method [14, 15]. Extensive skeletal deficiencies cannot be eliminated, but it provides a minimally invasive method of dentoalveolar compensation [14, 23, 52] in cases where orthognathic surgery is rejected [15, 25, 53, 54]. Moreover, premolar extractions can be avoided by uprighting mesially inclined posterior teeth for space obtainment.

## Conclusion

The MEAW technique appears to have significant therapeutic advantages and functions as a compensatory treatment modality in several types of malocclusions.

However, MEAW is not widely used beyond the Asian region and the DFA is based on skeletal patterns of the Asian population. Thus, further scientific investigations are needed in order to examine the transferability of the DFA to other ethnic groups more profoundly.

Concerning the deficient data basis of available literature and the weak scientific evidence, further studies are needed in order to take a closer look at aspects such as the potential effects on the periodontal tissue or the long-term stability.

## Data Availability

The datasets used and/or analysed during the study are available from the corresponding author on request.

## Abbreviations

MEAW: Multiloop Edgewise Archwire
MOAW: Multiloop Offset Archwire
SMOM: Sectional Modified Offset Archwire
DFA: Denture Frame Analysis
IA: Ideal Archwire
LDR: Load Deflection Rate
ODI: Overbite Depth Indicator
APDI: Anteroposterior Dysplasia Index
CF: Combination Factor
OP-MP: Occulsal Plane to Mandibular Plane
